# Computed tomography-guided puncture using a mobile application for a motion sensor-equipped smartphone

**DOI:** 10.1590/0100-3984.2018.0048

**Published:** 2019

**Authors:** Tiago Kojun Tibana, Renata Motta Grubert, Denise Maria Rissato Camilo, Edson Marchiori, Thiago Franchi Nunes

**Affiliations:** 1 Hospital Universitário Maria Aparecida Pedrossian da Universidade Federal de Mato Grosso do Sul (HUMAP-UFMS), Campo Grande, MS, Brazil.; 2 Universidade Federal do Rio de Janeiro (UFRJ), Rio de Janeiro, RJ, Brazil.

## INTRODUCTION

Computed tomography (CT)-guided puncture is one of the most widely used interventional radiology techniques, being employed in biopsies, drainage, and radiofrequency ablation procedures^(^^1^^-^^8^^)^. The technique varies greatly according to the imaging method chosen to guide the procedures. The disadvantage of using CT fluoroscopy as a real-time method is its tendency to increase exposure to ionizing radiation^(^^9^^,^^10^^)^. However, it has some real advantages, being able to provide real-time target locations, identify the positions of surrounding organs, and show the location of the puncture needle^(^^11^^)^. However, it is expensive to install and is therefore unavailable in many regions and cities. Therefore, the use of non-fluoroscopic puncture techniques continues to play an important role in current practice^(^^12^^)^.

The conventional puncture technique does not provide real-time guidance to track the needle and target location. The operators must advance the needle following the planned angle based on their own senses. The procedure can be stressful, due to the potential for puncture errors, which can affect vital organs and lead to complications. Therefore, to avoid such errors, it is necessary to follow a step-by-step process, with intermittent scanning of the region of interest to confirm the location of the needle each time the needle is advanced^(^^12^^)^, thus increasing the dose of radiation received by the patient.

To improve the convenience and accuracy of the conventional puncture technique, a smartphone application specifically developed to assist puncture guidance^(^^12^^)^, together with a radiopaque marker positioned on the skin, can be used.

## PROCEDURE

**Figure 1:**
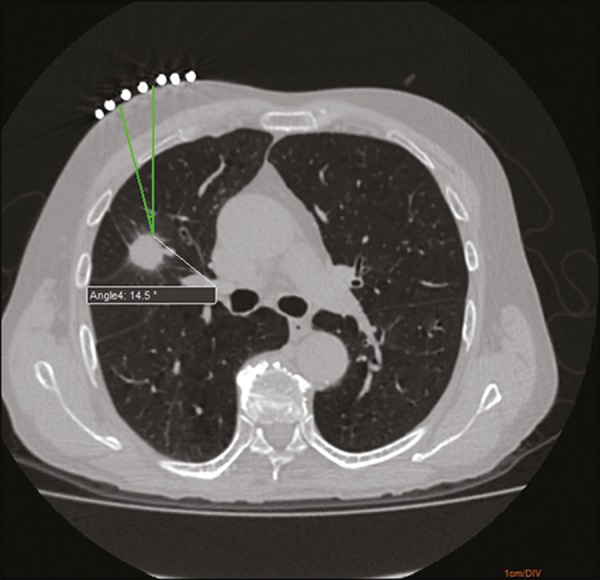
Axial CT of the chest, showing the puncture site planning, with angle calculation and marking of the skin between the radiopaque markers (arrow).

**Figure 2:**
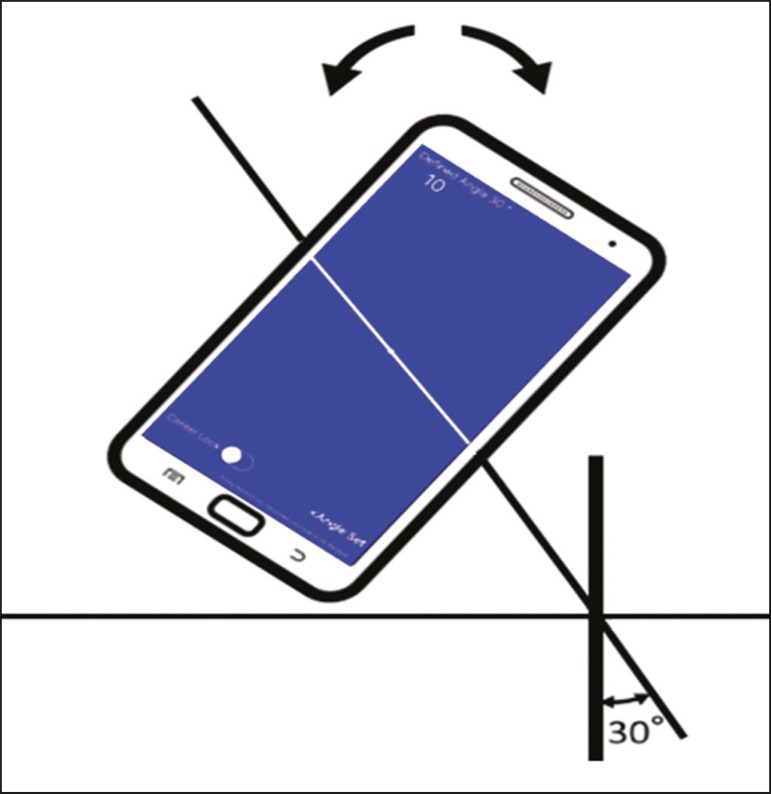
Illustration showing a hypothetical scenario of a puncture being made at a 30° angle with the viewfinder tilted down (blue screen).

**Figure 3:**
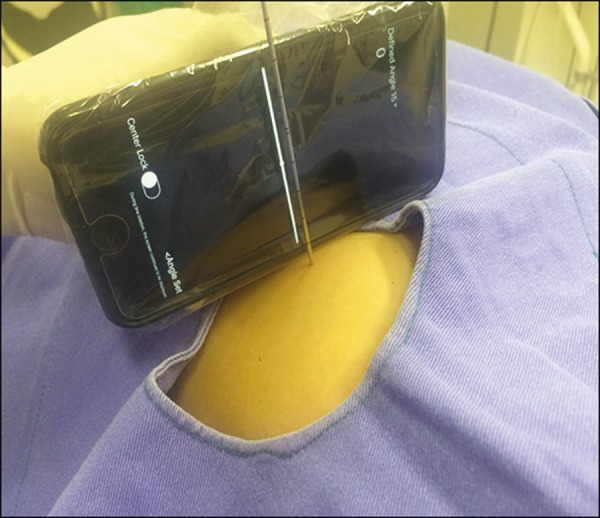
Insertion of the coaxial needle guided by the angle adjusted in the application.

**Figure 4:**
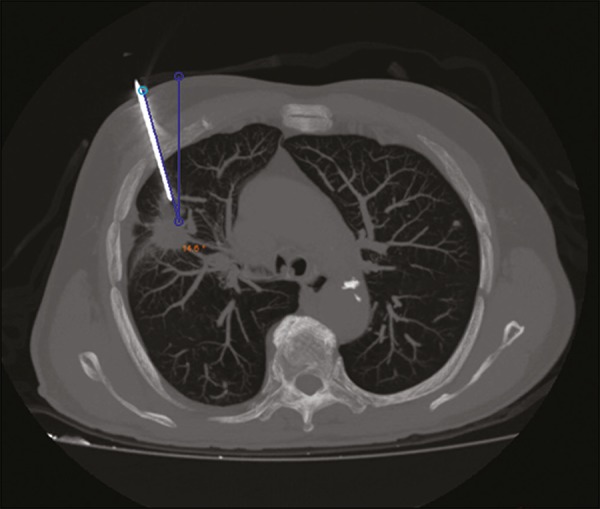
Axial MIP CT reconstruction showing correct insertion of the needle at the desired angle.

As in the conventional technique, the puncture site and angle are initially calculated by the CT workstation (Figure 1), the puncture site then being indicated with a mark on the skin of the patient. The smartphone is placed inside a sterile plastic bag to facilitate the sterilization process and ensure patient safety. Once the planned puncture angle has been entered into the application, a guide is displayed on the liquid crystal display. Motion sensors built into the device ensure that this orientation is constantly maintained at the desired angle regardless of the angle at which the device is held or whether or not it is in movement. The background color changes according to the screen angle: face up (pink), face down (blue) (Figure 2) and vertical (black). The center of rotation of the guide moves freely through the viewfinder and can be locked anywhere on the screen so that it is aligned with the desired puncture location. Finally, the smartphone is placed along the beam line marked on the surface of the skin and the puncture is performed by aligning the needle according to the orientation shown on the device (Figure 3). That prevents deviations, allowing the puncture to be made more precisely and at the appropriate angle (Figure 4).
